# Ecotoxicological effects of innovative wood protection treatments on freshwater bioindicator organisms

**DOI:** 10.1007/s11356-025-36796-4

**Published:** 2025-08-07

**Authors:** Nicoletta Nesto, Tihana Marčeta, Daniele Cassin, Francesco Acri, Andrea Pesce, Vanessa Moschino

**Affiliations:** Institute of Marine Sciences, National Research Council, Tesa 104 Arsenale, Castello 2737/F, Venice, 30122 Italy

**Keywords:** Ecotoxicology, Biomarker approach, Wood treatment, Impregnating agent, *Dreissena polymorpha*, *Theodoxus fluviatilis*

## Abstract

The protection of wooden structures used in aquatic environments is of paramount importance to prevent their attack by xylophagous organisms. With the aim of testing innovative and more eco-friendly wood treatments for the freshwater environments, we evaluated the possible ecotoxicological effects of *Picea abies* wood panels differently treated with copper-based formulation and thermal modification. The toxicity of the treatments was evaluated through exposure experiments by applying the biomarker approach in two freshwater bioindicators: the bivalve *Dreissena polymorpha* and the gastropod *Theodoxus fluviatilis*. The organisms were kept for 4 weeks in experimental tanks under semi-static conditions of the following: control (no panels), NTC (untreated panels), TT (thermally treated panels), and S-UC3 (panels treated with Cu-based formulation). Biomarkers in mussels exhibited no toxic effects attributable to NTC and TT conditions. The biological responses of the gastropods registered some stress signals not related to leaching process. Panels treated with the S-UC3 formulation caused 100% mortality of gastropods and damage effects in mussels. These responses were consistent with copper released in the water, which reached a concentration higher than 200 ppb. The study highlighted the need to improve the rinsing/maturation phase in wood panel impregnation protocols, to remove the Cu fraction easily released in freshwater environment. *T. fluviatilis* exhibited excessive sensitivity under microcosm conditions, indicating its unsuitability as a bioindicator in standardized aquarium exposure experiments.

**Graphical abstract:**

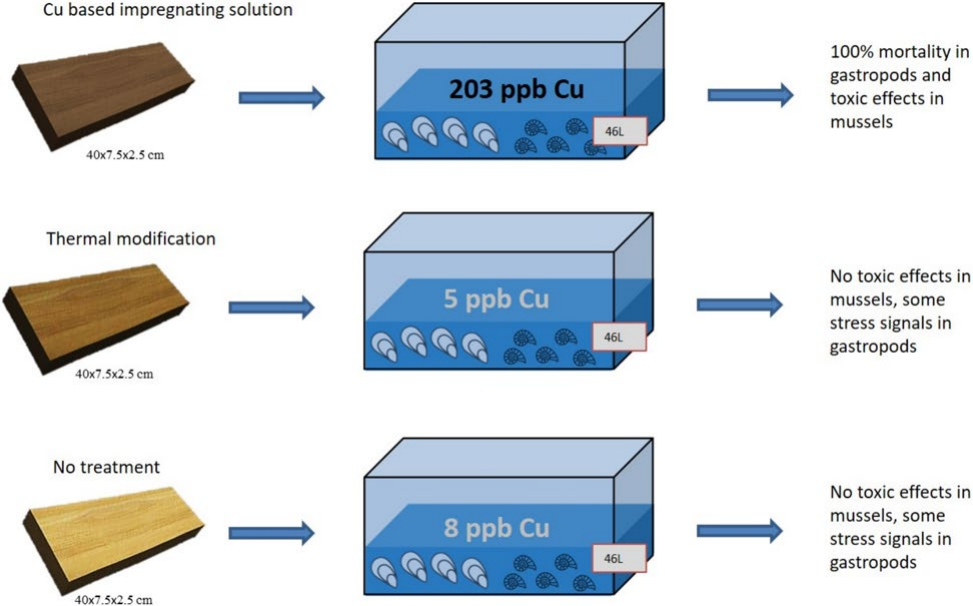

## Introduction

Wood is used as building material for a wide range of applications, including bridges, walkways, pontoons, and other structures placed within or close to waterways and wetlands, due to its structural, economic, environmental and aesthetic advantages (WWPI 2012). However, as a natural material, wood is subject to degradation phenomena and, if not adequately protected against the action of various biotic (fungi, insects, termites) and abiotic (temperature and humidity) decay factors, it loses its characteristics becoming structurally unusable (Marais et al. 2022). In the past, wood degradation was overcome by using highly durable hardwoods, such as cedar or cypress or by turning to tropical timbers. Today, due to environmental issues related to deforestation and the availability of the material, soft coniferous timbers are commonly used by the European wood construction industry. *Picea abies*, the Norway spruce, is the most widely used, due to its high availability and good workability. However, Norway spruce is not naturally durable, and application of protection agents is needed to comply with the use for outdoor structures (Caudullo et al. 2016).

Some substances naturally present in wood such as tannins, phlobaphenes, alkaloids, phenols, and polyphenes are capable of inhibiting insects and fungi colonization, providing the wood with some natural protection. Wood extracts and leachates from softwood conifers like pine and spruce have been demonstrated to contain bioactive compounds such as resin acids, phenolics, and terpenoids that pose ecotoxicological risks to aquatic organisms. For example, resin acids are toxic for fish at relatively low concentration range, i.e. 200–800 pg L^−1^, causing acute effects including respiratory distress and mortality (McFarlane and Clark 1988). Moreover, phenolic compounds and terpenoids inhibit microbial activity already at minimal concentrations < 300 mg L^−1^ (Metsämuuronen and Sirén, 2019). Libralato et al. (2011) assessed the toxicity of lignin and tannin on the marine alga *Phaeodactylum tricornutum*, reporting EC₅₀ values of 113.8 mg L^−1^ and 26.04 mg L^−1^ respectively, observing a morphological change of the algae fusiform shape at tannin concentrations ≥ 75 mg L^−1^ and < 185 mg L^−1^. Moreover, bioassays showed that tannic acid concentrations ranging from 0.06 to 2.0 mM have harmful effects on freshwater crustacean species such as *Chydorus sphaericus*, *Diaptomus castor*, and *Eucypris fuscata* (Pautou et al. 2000). Few data on environmental concentrations are available to properly understand the ecological relevance of these substances. Natural wood extract concentrations in freshwater ecosystems have been reported to be 8 mg/L for tannins (Earl et al. 2025), 8.5 μg/L for lignin (Reuter et al. 2017), 12 mg/L for resin acids near pulp mill effluent outfalls (Quinn et al. 2003), and 179 mg/L for phenolic compounds (Kumar and Pacha 2015). These outcomes suggest that the environmental concentrations of some of these substances might have ecotoxicological effects.

Despite these natural substances provide wood with some protection against xylophagous organisms, additional treatments are required before using it as construction material. There are mainly two methods for increasing wood durability: physical modifications (i.e., acetylation, furfurylation, thermal modification) and chemical protection using impregnating agents (Hill 2006; Gérardin 2016; Khademibami and Bobadilha 2022).

To increase wood durability, there are number of physical treatments aimed at altering the cell wall structure. The thermal treatment consists of a controlled pyrolysis process (*T* = 230/240 °C in the absence of oxygen), which allows the wood to become more compact and stable, and less hygroscopic and permeable. As a consequence, the wood results more resistant to xylophagous organisms, as the nutrients contained in the hemicelluloses are degraded by high temperatures, leading to the formation of degradation products such as acetic acid, methanol, formic acid, furfurals, and aldehydes that could be subject to leaching phenomena (Hill et al. 2021). To our knowledge, few studies have reported the ecotoxicological effects of heat-treated wood leachates on aquatic organisms. Esteves et al. (2011) conducted ecotoxicological assays on the bacteria (*Bacillus stearothermophilus*) using leachates from heat-treated pine wood and did not show any effect. A series of acute and chronic tests using leachates obtained from thermally modified wood (*Picea abies*) demonstrated that they have similar impacts to those of untreated wood. However, chronic tests using *Daphnia magna* resulted in high mortality of organisms when leachates derived from untreated and thermally modified wood samples were used directly, Conversely, a maturation period of at least 15 days produced less toxic leachates that allowed good levels of survival and offspring production (Picone et al. 2024).

Alternatively, the durability of wood may be chemically enhanced by impregnation with effective biocides. Copper-based biocides, in which copper compounds are combined with additives to increase the fixation capacity of copper, are the most commonly used for the impregnation of wood (Thaler and Humar 2014). In the past, mainly copper chromate arsenate (CCA) and/or creosote were used (Humar and Lesar 2013). Due to toxicity issues, the use of chromium-based wood preservatives has been significantly restricted or even banned in some EU countries since the implementation of the Biocides Directive (98/8/EC, 1998). The wood industry has therefore focused its efforts in developing alternative Cu-based wood preservative where Cr has been replaced by amines, mainly ethanolamine, in the preservative fixation process (Freeman and McIntyre 2008; Humar and Lesar 2013).

When exposed to environmental matrices, both thermally and chemically treated wood may be subject to leaching processes. The release of wood preservatives in the environment causes both a reduction in the service life of wooden structures and negative effects on environment and human health (Barbero-López et al. 2021). Leaching occurs both for bio-based compounds, such as pyrolysis distillates (Mohan et al. 2008), tannins (Sommerauer et al. 2019), and chitosans (Alfredsen et al. 2004) and for the Cu-based preservatives where Cr has been replaced by amines (Humar et al. 2001; Cooper and Ung 2009; Thaler and Humar 2014), but also for thermal modifications producing polysaccharide degradation products (Hill et al. 2021). Therefore, an ecotoxicological assessment, evaluating the possible effects of treated wood materials before their use in aquatic environment is of paramount importance for providing a judgement of environmental acceptability of the impregnating agents or physical treatments.

Freshwater ecosystems, whose conservation is regulated by Directive 2000/60/EC, are among the most diverse and dynamic environments on Earth as they play a crucial role in the biosphere, being biodiversity hotspots and providing essential resources for human survival and well-being (WWF 2023). Potential leaching of toxic compounds from artificial wooden structures such as pontoons, poles, bridges treated with chemical preservatives or thermally modified may represent sources of pollution, and a risk for aquatic organisms. Some studies have documented measurable leaching of copper, chromium, arsenic, zinc, and organic compounds from treated wood into adjacent soil, sediment, and water in natural environments such as wetlands, lakes, rivers, and estuaries (Forest Products Laboratory 2000; Wendt et al. 1996).

Typically, riverine systems with flowing water tend to have greater mobility and dispersal of preservative compounds, potentially leading not only to rapid downstream transport but also to their dilution. On the contrary, lakes and wetlands are often characterized by slower water movement and higher sedimentation, consequently leading to the accumulation of contaminants into sediments and biota, thus increasing long-term exposure and ecological risk (Weis and Weis 1996; Lebow 1996; Brooks 2000; Hingston et al. 2001). Concentrations of leachates might vary depending on proximity to treated wood, age of the structure, environmental conditions, and water exchange rates. Environmental concentration of copper typically ranges from 10 to 400 µg g^−1^ in sediments adjacent to treated structures (Weis and Weis 1992, 1996; Lebow and Tippie 2001), whereas water concentrations near new CCA-treated wood can reach up to 50 µg L^−1^ or more, but decrease over time due to leaching decline (Lebow 1996; Hingston et al. 2001). Chromium (Cr) and arsenic (As) were often found in sediments more than in water, as they were bound to fine particles, with levels in sediment near CCA-treated wood reaching 50–200 µg g^−1^ (Weis and Weis 1996; Brooks 2000; Hingston et al. 2001).

For a comprehensive assessment of the possible effects of a compound or a mixture of compounds in an aquatic environment, chemical analyses carried out in the various environmental matrices must be integrated with an assessment of their biological effects on organisms (Schuijt et al. 2021). In this context, a battery of biochemical, cellular, and physiological responses, commonly known as biomarkers, have been applied for the first time to evaluate the possible negative effect of wood treatments on the health status of bioindicators organisms. This study is part of a more complex research aiming at assessing the efficacy and the environmental compatibility of wood protection techniques and their possible impacts on aquatic environments, also through the application of ecotoxicological bioassays (Guarneri et al. 2021; Natali et al. 2023; Picone et al. 2024).

The biomarker approach, developed in the laboratory and subsequently validated in the environment since the 1980 s for the evaluation of the biological effects of priority pollutants, provides an integrated assessment of the ecosystem quality through the description of the state of well-being of the sentinel organisms (Bayne et al. 1985). The use of a biomarker battery, in which several biomarkers are measured simultaneously at different levels of biological organisation, allows to diagnose possible effects of contaminants to which bioindicator organisms have been exposed to and to predict the occurrence of long-term negative effects (Blaise and Gagné 2009; Dellali et al. 2022).

Biomarkers may be applied in both field-based monitoring and controlled laboratory exposure experiments, each showing distinctive advantages and limitations (Van der Oost et al. 2003; Galloway 2006). In field monitoring, biomarkers assess organism health in situ under real environmental conditions, capturing cumulative and synergic effects of multiple stressors. They are early-warning signals of sublethal stresses, valuable for long-term environmental surveillance and pollution effect tracking. The main strengths of their field applications are the ecological relevance and the ability to detect impacts over time and space, though interpretation is complicated by natural variability, unknown exposure histories, and confounding factors (Peakall 1994). In laboratory experiments, biomarkers are used under controlled conditions to establish cause-effect correlation concentration–response evaluation and the identification of ecotoxicological thresholds. Laboratory studies offer reproducibility and variable isolation, facilitating mechanistic understanding (Van der Oost et al. 2003; Galloway 2006).

In this study, the environmental sustainability of innovative wood treatments (a Cu-based biocide and a thermal treatment) was evaluated for freshwater habitats with the zebra mussel *Dreissena polym*o*rpha* and the nerite gastropod *Theodoxus fluviatilis*. The potential toxic effects of the two wood treatments were assessed in organisms exposed in static controlled laboratory condition, through the determination of a suite of biochemical and physiological biomarkers. The biological data were also evaluated in the light of the concentration of Cu and other metals found in the experimental tanks to verify the presence of possible leaching phenomena.

*D. polymorpha* is a typical sentinel species for freshwater environment both in Europe and in North America and commonly used in biomonitoring studies involving the application of the biomarker approach. This species shows many characteristics that make it suitable as biomonitor: it is sedentary, distributed in a variety of habitats, easy to sample, long-lived, and have a high filtration rate favouring the uptake and the bioconcentration of substances (Reeders et al. 1989; de Lafontaine et al. 2000; Faria et al. 2009; Palais et al. 2012; Binelli et al. 2015; Devin et al. 2023).

*T. fluviatilis* is a species highly abundant and widely distributed on stones in swift running rivers throughout Europe (Bunje and Lindberg 2007) and in the Baltic Sea (HELCOM 2012). It is considered very sensitive to disturbance according to AMBI (AZTI Marine Biotic Index) ecological group (Borja et al. 2000). This species has been already used only once as model organism in a biomonitoring study aimed at evaluating the effects of harbour contaminants in Sweden (Bighiu et al. 2017).

The aim of the present study is to evaluate the biological effects of the two wood treatments assessed in *D. polymorpha* and *T. fluviatilis* through the biomarker approach to provide a first insight of the potential toxicity before their use into freshwater environments. Lastly, as to our knowledge, this is the first study where *T. fluviatilis* has been used in exposure experiments under lab conditions, we also tested the suitability of this species as a possible sentinel organism for freshwater environments.

## Materials and methods

### Collection and treatment of the test organisms and exposure experiment design

Approximately 3000 adult specimens of the bivalve *D. polymorpha* and the gastropod *T. fluviatilis* were collected in two natural areas of the Veneto Region (Italy) in October 2021: a site located in the Northern Garda Lake (45°52′54.49″N, 10°50′18.56″E) and a site located in the Limbraga river, a tributary of the Sile River (45°39′44.54″N, 12°16′17.97″E), respectively. For both areas, the ecological and chemical status of the water bodies were considered good in 2020 under the monitoring of the Water Framework Directive (Provincia Autonoma di Trento 2020; ARPAV 2021). Organisms were maintained 2 weeks before the experiments in 30-L aquaria filled with aerated dechlorinated tap water at temperature 20 ± 0.5 °C with a 14 h:10 h light/dark cycle for acclimatization. During the acclimatization period, organisms were fed with Perla Larva Proactiv (®Hendrix) and aquatic macrophytes every second day, and half water was renewed every 2 days, particularly the day after feeding. Check for mortality were daily carried out and moribund or dead individuals were promptly removed.

The wooden panels to be tested were prepared and supplied by Silvaprodukt d.o.o. (Ljubljana, Slovejia) according to “Use Class 3” (UC3) contained in the EN 335 standard (CEN European Committee for Standardization 2013). Wooden panels were impregnated with a formulation of Silvanolin® having a copper concentration of 0.25% according to a vacuum-pressure procedure (30 min vacuum at 10 kPa; 3 h pressure at 900 kPa; 15 min vacuum at 20 kPa) and subjected to a conditioning procedure over a month at a temperature of 20 °C and 70% relative humidity (Humar et al. 2008).

For the thermal modification, the wooden panels were isothermally treated for 3 h at 190, 200, 210, 220, and 230 °C, respectively, according to the Silvapro® commercial procedure (Rep and Pohleven 2001).

**Fig. 1:**
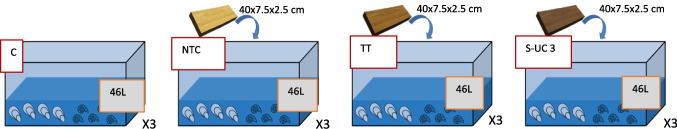
Scheme of the experimental tank setup. Mussels and gastropods were exposed for 4 weeks to the different experimental conditions: C (no wood panel), NTC (not treated wood panel); TT (thermically treated wood panel); S-UC3 (wood panel treated with 0.25% Cu impregnating solution). Three tanks were set up for each experimental condition (replicates)

After the acclimatisation period, approximately 200 mussels and 250 gastropods were placed randomly in each experimental tank and maintained for 4 weeks under four experimental conditions: control (C, no wood panels), untreated *P. abies* wood panels (NTC), thermally treated *P. abies* wood panels (TT), and *P. abies* wood panels treated with impregnating solution containing 0.25% Cu (S-UC3). Three tanks (i.e., replicates) containing 46 L of freshwater were set up for each experimental condition. In each tank, excluding controls, one wood panel (40.0 × 7.5 × 2.5 cm) was placed at the bottom of the tank by two pebbles previously washed with distilled water (Fig. 1). Wood panel surface/volume ratio was 0.018 cm^2^ cm^−3^. Given the absence of specific protocols, this surface/volume ratio, used also for the experiments performed in seawater, was chosen based on the results obtained in acute toxicity tests aimed at assessing the survival capacity of the crustacean *Acartia* (*Acanthacartia*) *tonsa* when exposed to the same impregnating solution containing 0.25% Cu (Volpi Ghirardini et al. 2022).

Tanks were provided with oxygen and *D. polymorpha* and *T. fluviatilis* specimens were fed every 2 days with Perla Larva Proactiv (®Hendrix) and aquatic macrophytes collected along the margins of the Sile River, respectively. A quart of water was renewed every 2 days, particularly the day after feeding to avoid nitrate increase. Water samples were collected during the water changes for chemical analysis. During the experiment, the organisms were kept at a temperature of 20 ± 0.5 °C, with a 14 h:10 h light/dark cycle.

### Chemical analyses

The samples for the analyses of dissolved nutrients, N-NH_3_ (ammonia), N-NO_2_ (nitrite), N-NO_3_ (nitrate), and P-PO_4_ (phosphate) were filtered on Whatman GF/F glass fiber filters (porosity = 0.7 µm) and analyzed with an EasyChem Plus analyser, manufactured by SYSTEA S.p.A. following Grasshoff et al. (1983) and Doane and Horwath (2003):

- N-NH_3_: formation of indophenol blue and reading at 630 nm (DL = 0.05 µM);
- N-NO_2_: diazotization and formation of a red nitrogenous compound read at 546 nm (DL = 0.01 µM);
- N-NO_3_: diazotization and formation of a red nitrogenous compound read at 546 nm after quantitative reduction of nitrate to nitrite by vanadium (III). Finally, a correction has to be made for nitrite present in the sample (DL = 0.10 µM);
- P-PO_4_: formation of a blue phosphomolybdic complex and subsequent reading at 880 nm (DL = 0.02 µM).

In the results the sum of ammonia, nitrite and nitrate is indicated as DIN, dissolved inorganic nitrogen.

The concentration of Cu and other metals (Hg, Al, Ba, Cd, Cr, Fe, Mn, Ni, Pb, and Zn) was determined by inductively coupled plasma atomic emission spectrometry in integrated water samples collected from each experimental condition after 2, 10, 17, and 27 days from the beginning of the experiment to assess the possible release of inorganic compounds from the treated wood panels into the water.

In the laboratory, the water samples were acidified by addition of nitric acid to a pH < 2, appropriately diluted and then directly analyzed by inductively coupled plasma (ICP-AES) (USEPA 1994). Calibration for ICP- AES analysis was achieved with prepared external standards via the standard curve approach. Full calibration was performed after every set of 48 samples. The method detection limit for element analysis was defined as three times the standard deviation of ten blank measurements.

### Determination of biomarkers

The possible toxic effects of different wood panel treatments were assessed in the two bioindicator species through the determination of a suite of biomarkers applied at different level of biological organisation. At the biochemical level, biological responses were assessed in terms of the activity of some antioxidant/detoxifying enzymes such as catalase (CAT) and glutathione-transferase (GST) involved in the elimination of free radicals, according to methods described in Wojtal-Frankiewicz et al. (2014) and Habig and Jakoby (1981), respectively. The activity of the enzyme acetylcholinesterase (AChE), involved in the transmission of electric impulses at synaptic level, was determined according to Binelli et al. (2005). The amount of metallothionein (MT), cytosolic proteins involved in metal detoxification processes, was assessed according to the spectrophotometric methods described in Kimura et al. (1979) and Viarengo et al. (1997).

Due to the small size of the mollusc species used, biochemical biomarkers were determined on the whole soft tissue. All biochemical determinations were referred to the total protein content measured according to Bradford (1976). Each determination was performed on two to three pools per experimental tank, each pool consisting of at least five to ten organisms depending on the species.

To evaluate the general physiological state of the zebra mussels, the condition index, providing information on the organisms’ body mass, was determined. The condition index [IC = (soft tissue dry weight/shell dry weight) × 100] was assessed on four individuals from each tank (12 organisms per experimental condition) according to Crosby and Gale (1990). Furthermore, during the 4-week experiment, mortality was daily monitored for both species, and dead organisms were promptly removed from the tanks.

### Statistical analysis

Significant differences among experimental conditions were assessed for each parameter by means of a linear mixed model with the tank as a random effect and followed by Tukey’s post-hoc correction using the R programme (R Core Team 2020). For all statistical analyses, the significance level was set at* p* < 0.05.

## Results

### Chemical analyses

The concentrations of metals determined in the water samples collected 2, 10, 17, and 27 days after the beginning of the experiment are shown in Table 1. In general, the concentrations of Hg, Cd, Fe, and Ni were below the instrumental detection limit in all the experimental tanks. The concentrations of Al, Ba, Cr, Mn, Pb, and Zn were low and constant over time. Cu concentrations were low in all experimental conditions except for the tanks containing the panels treated with the 0.25% Cu impregnating solution (S-UC3). Specifically, during the experiment the values ranged between 3 and 7 ppb in the control tanks (C), 3 and 8 ppb in the untreated panel tanks (NTC), and 4 and 5 ppb in the thermally treated panel tanks (TT). In S-UC3, Cu concentration showed a value of 156 ppb after 2 days from the beginning of the experiment and reached its highest value of 203 ppb after 10 days, before gradually decreasing to 129 ppb after 27 days.

**Table 1:** Metal concentrations (ppb) in water periodically sampled at the different experimental conditions: C (no wood panel), NTC (not treated wood panel); TT (thermically treated wood panel); S-UC3 (wood panel treated with 0.25%Cu impregnating solution)

		Hg	Al	Ba	Cd	Cr	**Cu**	Fe	Mn	Ni	Pb	Zn
Experimental condition	Day	*ppb*	*ppb*	*ppb*	*ppb*	*ppb*	***ppb***	*ppb*	*ppb*	*ppb*	*ppb*	*ppb*
C	2	< d.l	78	37	< d.l	1	**3**	6	0	< d.l	3	8
	10	< d.l	28	41	< d.l	1	**7**	< d.l	1	< d.l	4	7
	17	< d.l	30	43	< d.l	1	**6**	< d.l	1	< d.l	2	8
	27	< d.l	135	47	< d.l	1	**6**	17	1	< d.l	4	15
NTC	2	< d.l	17	26	< d.l	1	**3**	< d.l	2	< d.l	3	6
	10	< d.l	23	38	< d.l	1	**8**	< d.l	1	< d.l	4	6
	17	< d.l	23	39	< d.l	1	**7**	< d.l	1	< d.l	2	7
	27	< d.l	32	47	< d.l	1	**4**	4	2	1	3	12
TT	2	< d.l	30	34	< d.l	1	**5**	< d.l	2	< d.l	4	5
	10	< d.l	27	33	< d.l	1	**4**	1	2	< d.l	4	6
	17	< d.l	28	41	< d.l	1	**5**	2	1	< d.l	3	14
	27	< d.l	34	44	< d.l	1	**5**	2	1	< d.l	2	8
S-UC3	2	< d.l	24	36	< d.l	1	**156**	< d.l	12	< d.l	3	8
	10	< d.l	45	44	< d.l	1	**203**	5	9	1	4	15
	17	< d.l	37	43	< d.l	1	**173**	1	7	< d.l	3	14
	27	< d.l	33	44	< d.l	2	**129**	3	4	< d.l	3	10
*d.l*		*0.1*	*10*	*1*	*1*	*1*	*2*	*1*	*1*	*1*	*2*	*5*
*L.D. 31/01*		*1*	*200*	*-*	*5*	*50*	*1000*	*200*	*50*	*20*	*10–25*	*-*

*d.l.* detection limits

L.D. 31/01 = limits imposed by Legislative Decree 31/01—Implementation of Directive 98/83/EC on the quality of water intended for human consumption

The nutrient levels determined in water samples at the end of the experiment are shown in Table 2. Ammonium, nitrite, nitrate, and phosphate concentrations resulted similar in the different experimental conditions, showing values ranging from 1.7 to 4.1 µM for N-NH_3_, from 23.9 to 60.7 µM for N-NO_2_, from 592.7 to 651.4 µM for N-NO_3_, and from 6.8 to 8.8 µM for P-PO_4_.

**Table 2:** Nutrient concentrations (µM) in water samples taken at the end of the experiment at the different experimental conditions: C (no wood panel), NTC (not treated wood panel); TT (thermically treated wood panel); S-UC3 (wood panel treated with 0.25%Cu impregnating solution)

	N-NH3		N-NO2		N-NO3		DIN*		P-PO4	
	mean	sd	mean	sd	mean	sd	mean	sd	mean	sd
C	4.1	3.4	29.5	8.8	651.4	62.1	684.9	62.0	8.8	1.36
NTC	2.0	0.9	23.9	15.9	622.2	22.4	648.0	37.9	7.2	1.59
TT	3.2	1.2	60.7	6.1	592.7	33.4	656.5	28.3	8.1	0.704
S-UC3	1.7	0.9	40.4	44.5	622.5	49.2	664.5	29.5	6.8	0.99

*sd* standard deviation

^*^Dissolved inorganic nitrogen

### Biological analyses

**Fig. 2:**
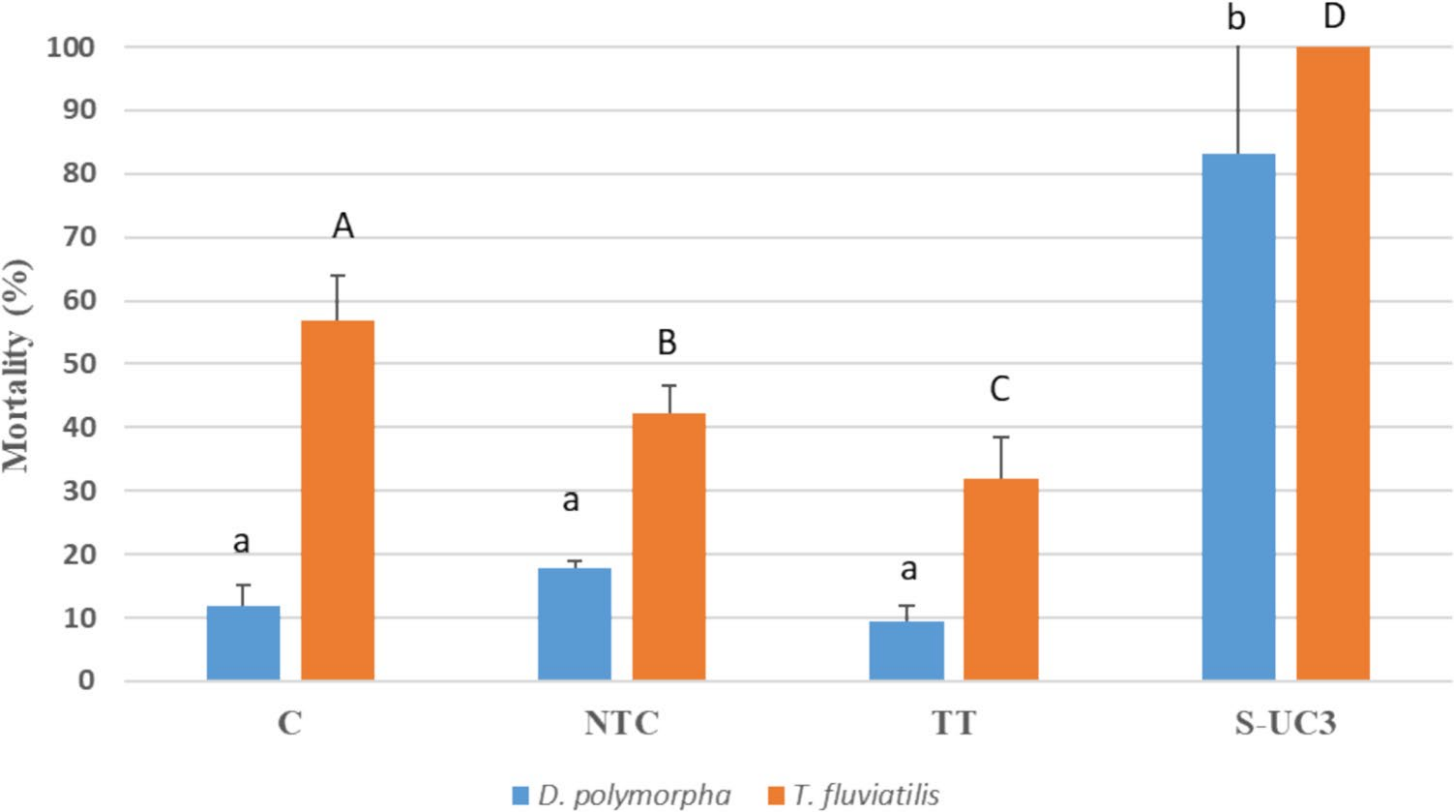
Mortality (mean ± sd, *n* = 3) of *D. polymorpha* and *T. fluviatilis* exposed for 4 weeks at: C (no wood panel), NTC (not treated wood panel), TT (thermically treated wood panel), S-UC3 (wood panel treated with 0.25% Cu impregnating solution). Statistical differences (*p* < 0.05) among experimental conditions (according to the Tukey post-hoc correction results) are presented with lower case letters (a, b) for *D. polymorpha* and with capital letters (A-D) for *T. fluviatilis*

The mortality registered for *D. polymorpha* and *T. fluviatilis* exposed to different experimental conditions is shown in Fig. 2. Mussels exposed to the panels treated with the S-UC3 formulation reached mortality levels of 83.17 ± 17.96% at the end of the experiment. These values resulted significantly higher than those reached by organisms exposed to the other three experimental conditions (C, NTC, and TT). Mortality recorded in mussels exposed to control condition (C), untreated panels (NTC), and thermally treated panels (TT) showed values of 11.83 ± 3.33%, 18 ± 1%, and 9.50 ± 2.29%, respectively. Gastropods showed higher mortality levels than mussels. All gastropods exposed to S-UC3 panels died after 2-day exposure and for this reason it was not possible to proceed with the analysis of other biological parameters for this sample. The values of mortality resulted significantly different among all experimental conditions, reaching values of 32.04 ± 6.32% for sample exposed to TT wood panel, 56.90 ± 6.91% for C samples, and 42.20 ± 4.25% for sample exposed to NTC conditions.

**Fig. 3:**
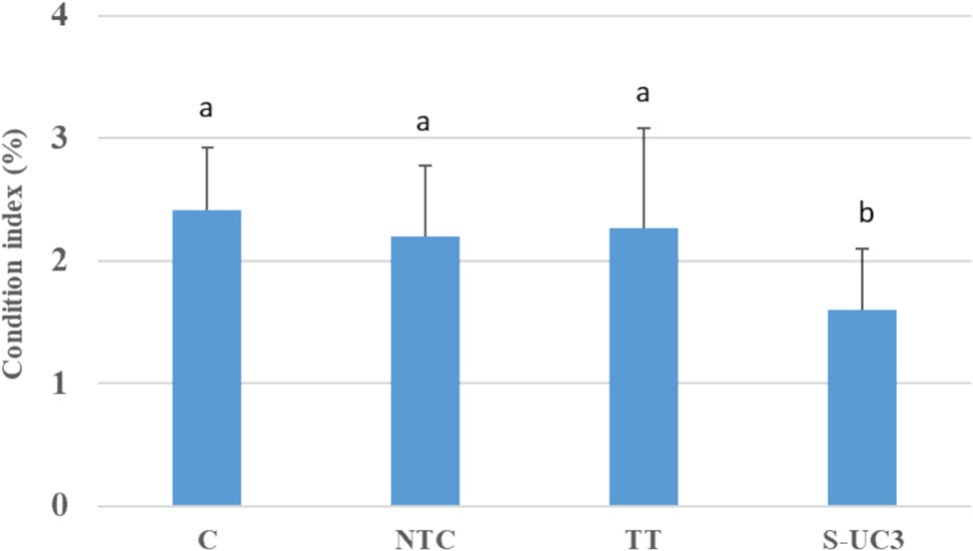
Condition index (mean ± sd, *n* = 3) measured in *D. polymorpha* exposed for 4 weeks at: C (no wood panel), NTC (not treated wood panel), TT (thermically treated wood panel), S-UC3 (wood panel treated with 0.25% Cu impregnating solution). Statistical differences (*p* < 0.05) among experimental conditions (according to the Tukey post hoc correction results) are presented with lower case letters (a, b)

The condition index showed significantly lower values in mussels exposed to S-UC3 panels (1.60 ± 0.50) compared to the samples from the remaining experimental conditions (C 2.42 ± 0.50, NTC 2.20 ± 0.58, TT 2.42 ± 0.50) (Fig. 3).

**Fig. 4:**
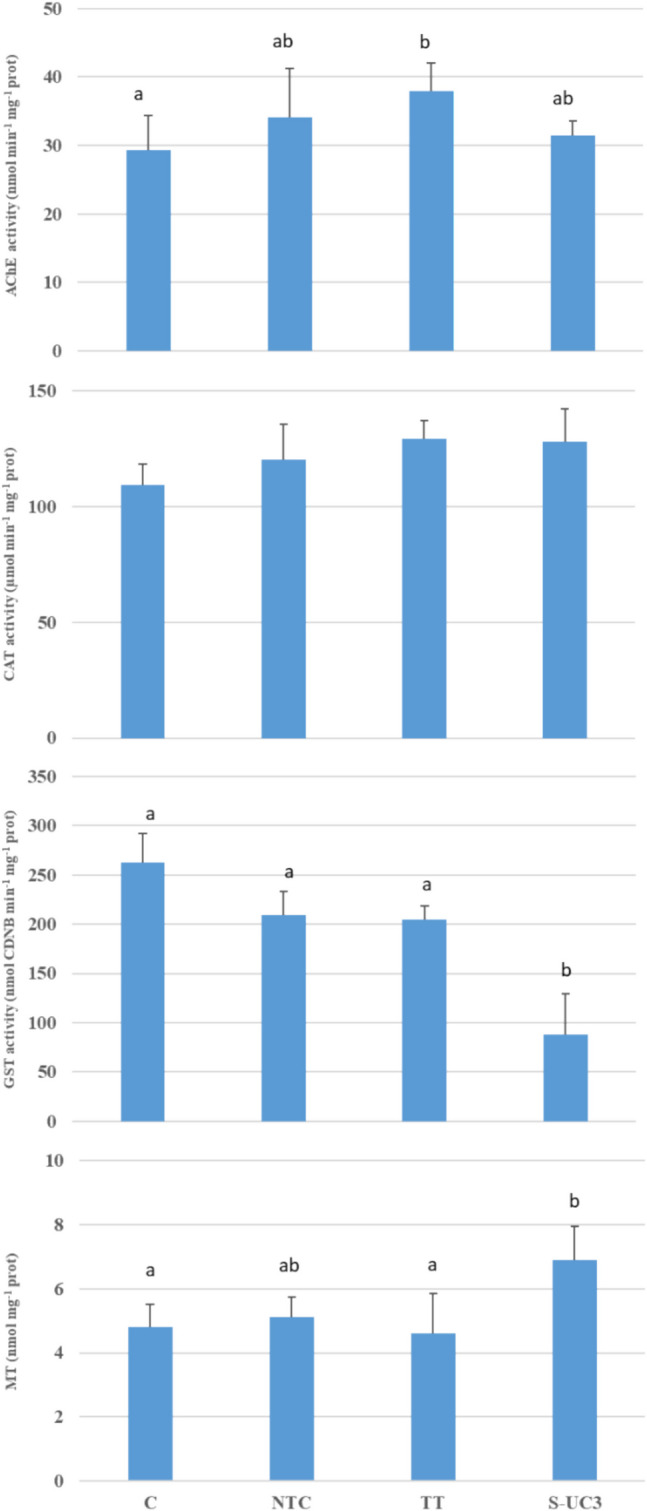
Acetyl cholinesterase activity (AChE), catalase activity (CAT), glutathione S tranferase activity (GST), and metallotioneins content (mean ± sd, *n* = 3) measured in *D. polymorpha* exposed for four weeks at: C (no wood panel), NTC (not treated wood panel), TT (thermically treated wood panel), S-UC3 (wood panel treated with 0.25% Cu impregnating solution). Statistical differences (*p* < 0.05) among experimental conditions (according to the Tukey post hoc correction results) are presented with lower case letters (a, b)

The results from the biomarker analysis carried out in *D. polymorpha* samples are reported in Fig. 4.

AChE activity resulted significantly lower in control samples (29.32 ± 0.5 nmol min^−1^ mg^−1^ protein) than in TT samples (37.91 ± 4.19 nmol min^−1^ mg^−1^ protein). The activity measured in S-UC3 and NTC samples showed similar and intermediate values equal to 31.40 ± 2.13 and 34.11 ± 7.17 nmol min^−1^ mg^−1^ protein, respectively.

As regards the activities of the antioxidant enzymes, CAT values were lower in C sample (109.43 ± 8.86 µmol min^−1^ mg^−1^ protein) with respect to the others, although the differences were not statistically significant. Highest CAT activity (129.04 ± 7.82 µmol min^−1^ mg^−1^ protein) was found in S-UC3 sample. GST activity resulted significantly depressed in S-UC3 sample (88.03 ± 40.99 nmol CDNB min^−1^ mg^−1^ protein) with respect to the other samples, which showed similar values, ranging from 205.09 ± 13.4 in TT to 262.63 ± 29.63 nmol CDNB min^−1^ mg^−1^ protein found in C.

The MT content resulted significantly higher in S-UC3 samples (6.89 ± 1.05 nmol mg^−1^ protein) compared to C (4.81 ± 0.71 nmol mg^−1^ protein) and TT (4.61 ± 1.25 nmol mg^−1^ protein) samples.

**Fig. 5:**
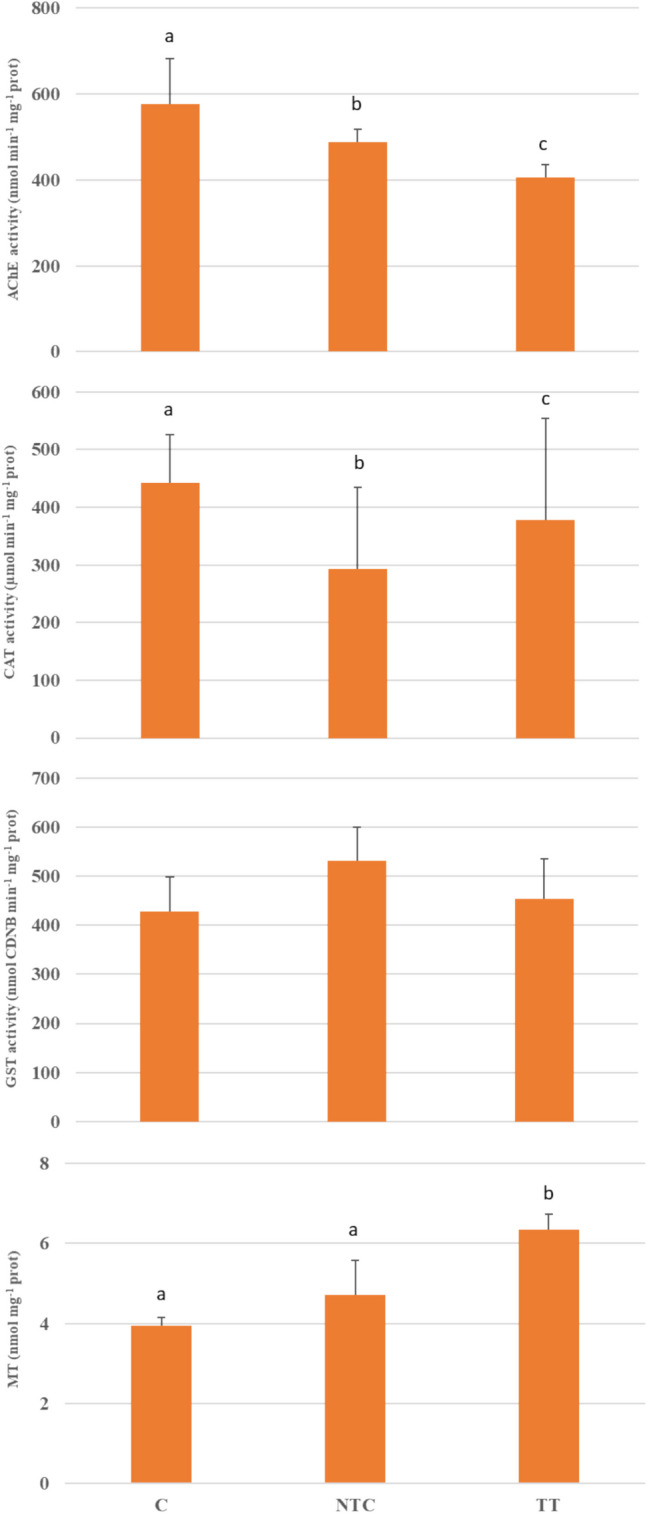
Acetyl cholinesterase activity (AChE), catalase activity (CAT), glutathione S tranferase activity (GST), and metallotioneins content (mean ± sd, *n* = 3) measured in *T. fluviatilis* exposed for four weeks to the different experimental conditions: C (no wood panel), NTC (not treated wood panel), TT (thermically treated wood panel), S-UC3 (wood panel treated with 0.25% Cu impregnating solution). Statistical differences (*p* < 0.05) among experimental conditions (according to the Tukey post hoc correction results) are presented with lower case letters (a–c)

The results from the biomarker analysis carried out in *T. fluviatilis* samples are reported in Fig. 5.

The AChE activity was statistically different among all experimental conditions and in particular decreased in both NTC (489.12 ± 29.81 nmol min^−1^ mg^−1^ protein) and TT (406.66 ± 28.36 nmol min^−1^ mg^−1^ protein) samples compared to C (577.42 ± 105.23 nmol min^−1^ mg^−1^ protein).

CAT activity was statistically different among all experimental conditions with significantly lower values in NTC (293.57 ± 140.93 µmol min^−1^ mg^−1^ protein) and TT (377.77 ± 175.86 µmol min^−1^ mg^−1^ protein) samples, compared to C sample (442.92 ± 82.27 µmol min^−1^ mg^−1^ protein).

GST activity showed higher values in NTC (531.12 ± 69.38 nmol CDNB min^−1^ mg^−1^ protein) and TT (454.70 ± 81.51 nmol CDNB min^−1^ mg^−1^ protein) samples than in C (428.65 ± 69.86 nmol CDNB min^−1^ mg^−1^ protein) and no statistical differences were found among experimental conditions.

The MT content resulted significantly higher in TT sample (6.35 ± 0.39 nmol mg^−1^ protein) than both C and NTC samples, which showed statistically similar values (3.95 ± 0.21 and 4.71 ± 0.86 nmol mg^−1^ protein, respectively).

## Discussion

In this study, the biomarker approach was used to assess the effects of both physical and chemical treatments applied to *P. abies* wood panels in a freshwater environment. In particular, we designed a 4-week exposure experiment in order to evaluate the ecological effects of the release of potentially toxic substances in the water using two bioindicator species: the zebra mussel *D. polymorpha* commonly used in environmental quality assessment (de Lafontaine et al. 2000; Faria et al. 2009) and the river nerite *T. fluviatilis* for the first time as bioindicator to evaluate potential ecotoxicological effects through exposure experiments.

Analyses of metals determined in the water samples showed that wood panels treated with the S-UC3 formulation caused a marked release of Cu into the water, with the highest concentration observed after 10 days (203 ppb), for then decreasing at the end of the experiment. This observation is in agreement with the results of other studies on leaching dynamics of copper-ethanolamine treated wood, indicating that leaching is greater in its initial phase (Thaler and Humar 2014).

Copper (Cu) is an essential micronutrient, as it is a component of various enzymes. However, Cu can also be toxic to aquatic organisms at environmentally relevant concentrations. Rajalakshmi and Mohandas (2005) investigated acid phosphatase activity pattern in the gills and hepatopancreas of the freshwater mussel *Lamellidens corrianus* exposed to copper concentrations varying from 100 to 400 µg L^−1^. Their findings indicated a higher enzyme activity in gills as a short-term response to metal toxicity, and inhibition of enzyme synthesis in the hepatopancreas. The filtration rate and survival of zebra mussels (*Dreissena polymorpha*) were investigated during chronic exposure to Cu concentrations up to 100 µg L^−1^, resulting in a EC50 of 43 µg L^−1^ (Kraak et al. 1992). It is worth noting that the Cu concentrations observed in this study over time in the S-UC3 experimental tanks were highly above the levels causing adverse effects to aquatic life (Mebane 2023). As for the Environmental Quality Standard under the WFD, 21 EU countries have set threashold values for Cu in freshwaters, varying between 0.5 and 120 µg Cu L^−1^ (European Environment Agency 2022). Additionally, Wilson et al. (2023) confirmed the concentration of 1 µg Cu L^−1^ as Environmental Quality Standard based on bioavailability.

The copper leached from S-UC3 formulation resulted in 100% mortality of the *T. fluviatilis* and in more than 80% mortality of *D. polymorpha*, coupled with the presence of some stress signals in the surviving mussels. The mortality recorded in S-UC3 conditions could also be caused by other synergic unforeseen factors that also lead to the mortality of control organisms. This result confirms the findings of Picone and co-authors (2024) who reported that copper and Cu-complexes were identified as the main contaminants of concern in leachates obtained from Norway spruce specimens treated with the same Cu-based preservative solution. Moreover, in the same study, the toxicity of the leachates was demonstrated through the application of a tiered Integrated Testing Strategy (ITS) (Picone et al. 2024). The Cu leaching in freshwater medium may be due to the use of ethanolamine as fixative agent in the preservative formulation (Humar et al. 2001). Although few studies focused on copper leaching mechanisms from copper-ethanolamine treated wood, the results available so far indicated that ethanolamine is not particularly effective to fixate Cu (Cooper and Ung 2009; Thaler and Humar 2014). The toxicity of S-UC3 treatment was demonstrated in ecotoxicological tests, addressing acute effects on *Aliivibrio fischeri*, *Raphidocelis subcapitata*, and *Daphnia magna* (Picone et al. 2024), and causing modifications on microbial biofilm formation in wood panels exposed in the Grado Lagoon (Natali et al. 2023).

In the present study, the mortality values registered in mussel samples exposed to both TT and NTC wood panels were low and similar to C sample, suggesting that NTC panels and TT wood treatment were generally well tolerated by *D. polymorpha*. Conversely, gastropod samples showed significantly higher mortality values than mussels in all experimental conditions, providing evidence of a stress syndrome experienced by *T. fluviatilis*, thus resulting more sensitive than *D. polymorpha*. The higher tolerance to copper concentrations of zebra mussels was also observed by Claudi et al. (2023) in comparison to quagga mussels (*Dreissena rostriformis bugensis*). The authors found that zebra mussels accumulated more than twice copper than quaggas, suggesting that numerous physiological aspects might play a role to explain this finding, although the toxicity mechanisms still need to be clarified. Contrary to zebra mussels, in gastropods, the mortality data suggest the presence of a weak physiological condition also in the reference organisms (C and NTC). The high mortality rates recorded also in the control organisms might indicate that laboratory conditions prolonged for long periods are not suitable for this species, making specimens even more sensitive to treatments. Moreover, the higher survival rates recorded in TT and NTC samples compared to C organisms could be attributed to the presence of wood not impregnated with chemicals in tanks, which increased the available surface for biofilm growth, and *T. fluviatilis* typically grazes on hard benthic surfaces, including submerged wood (Orton and Sibley 1990; Abdallah 2015).

*T. fluviatilis* is considered very sensitive to disturbance also according to AMBI ecological group (Borja et al. 2000) and the results of the overall physiological state observed in this study may be considered, although preliminary, as indicative of its unsuitability as biomonitor, at least when used in laboratory conditions. To verify if the trophic state of the water medium would have influenced the biological responses of the biomonitor organisms, the nutrient concentrations were analysed in the experimental tanks. The levels of N-NH_3_, N-NO_2_, N-NO_3_, DIN, and P-PO_4_ determined in water at the end of the exposure experiment was overall similar among all experimental conditions, ruling out the possibility that the organisms had been fed differently depending on the tanks or that altered concentrations of ammonia, nitrate, nitrite, and phosphate could have impacted the fitness of the organisms.

The condition index (CI), giving an indication of the amount of body mass relative to the shell, is a parameter routinely used to assess the general health status of the organism (Crosby and Gale 1990). CI showed for zebra mussels significantly lower values in organisms exposed to the S-UC3 treatment than in all other samples, revealing their low physiological conditions, likely related to the Cu release in the water from S-UC3 wood panels.

Considering the biomarker analyses, no significative inhibition of the AChE activity was evidenced in *D. polymorpha* exposed to S-UC3 and TT wood panels when compared to NTC and reference samples, whereas in *T. fluviatilis* both NTC and TT samples showed significantly lower AChE activities than C sample. AChE enzyme is implicated in the proper transmission of electrical signals at the level of synaptic membranes, and it is usually considered an indicative parameter of exposure to organic contaminants (such as organophosphoric and carbamate) (UNEP/MAP-MEDPOL 2021). The AChE levels observed in *D. polymorpha* samples resulted comparable with those determined in organisms collected in pristine areas along the Lake Balaton in Hungary (Farkas et al. 2017) and Lawrence River in Canada (de Lafontaine et al. 2000). These findings coupled with the absence of statistical difference in the enzyme activity determined in mussel samples suggest the absence of a neurotoxic effect related to both two treatments (chemical and physical) in *D. polymorpha*. In *T. fluviatilis*, the levels of AChE activity resulted generally higher than *D. polymorpha* and significantly depressed in sample exposed to both NTC and TT wood panels in comparison with control sample. The presence of neurotoxic effects in *T. fluviatilis* was possibly due to the release into the water of both natural substances from NTC, such as phenols and aldehydes (Libralato et al. 2007), or thermal degradation products from TT wood panels, such as formic acid and acetic acid (Hill et al. 2021)*.*

In *D. polymorpha*, the antioxidant activity of CAT was similar between controls and treated samples, whereas GST activity was significantly reduced in the sample exposed to S-UC3. These two enzymes are involved in detoxifying cells processes from reactive oxygen species (oxyradicals) and, generally, their activities tend to increase upon exposure to contaminants. However, CAT and GST enzymes are characterized by a bell-shaped dose response curve, and their activities are therefore inhibited above a certain threshold of toxic substances exposure (Viarengo et al. 2007). For this reason, the interpretation of the results for these biomarkers may be complex. Although the GST levels suggest the possible presence of stress signals, especially in S-UC3 sample, where a marked inhibition of the enzymatic activity occurs, the CAT levels recorded in all samples could be considered high if compare to the activity determined in *D. polymorpha* sampled in two reference areas: Rybinsk Reservoir (Russia) (76.90 ± 3.63 nmol min^−1^ mg^−1^ protein) (Klimova et al. 2019), and Rouge River, Michigan (USA) (4–8 µmol min^−1^ mg^−1^ protein) (Nowicki and Kashian 2018). However, the enzymatic activity is lower than that one observed in the control organisms by Parolini et al. (2010), with mean values around 200 µmol min^−1^ mg^−1^ protein, and similar to the values in the study of Riva et al. (2010), with control organisms showing CAT activity of about 70 µmol min^−1^ mg^−1^ protein. In *T. fluviatilis*, the activities of the antioxidant enzymes were generally higher than *D. polymorpha*. No significative differences were recorded among C, NTC, and TT samples for GST, whereas a statistically lower value was registered in NTC sample for CAT when compared to control sample, suggesting the possible presence of oxidative stress.

Metallothioneins are cytoplasmic proteins characterised by a high cysteine content that makes them excellent chelating agents. They are involved in the intracellular regulation of essential metals (Cu, Zn) and are induced by the presence of metals. Moreover, they play an important role in the neutralisation of toxic metals (Hg, Cd) and in the protection of metal-induced oxidative stress (Viarengo et al. 1997; Vergani et al. 2005). In mussels, MT concentrations were similar in controls and in samples exposed to the NTC and TT panels, whereas they were significantly higher in sample exposed to S-UC3, suggesting the presence of induction phenomena aimed at sequestering Cu excess at the cellular level. The MT levels registered in S-UC3 samples were higher than those determined in the same species sampled in harbor and industrial zones along the St Lawrence River (Canada) contaminated by metals and POPs, evidencing that MT levels exceeding 6 nmol mg^−1^ protein may be considered indicative of the presence of metal toxicity for this species (de Lafontaine et al. 2000). In *T. fluviatilis*, the concentration of MTs resulted similar to those registered in *D. polymorpha*. The significantly induction of MTs in TT and NTC samples may due, also for this biomarkers, to the presence in the medium of toxic compounds such as formic and acetic acids released by TT panels (Hill et al. 2021) or phenols, lignin, and tannins released by NTC panels (Libralato et al. 2007, 2011; Pessala et al. 2010; Alvarez et al. 2016; Picone et al. 2024), which may have determined the induction of these proteins, known to play an antioxidant role in inflammatory processes as well (Coyle et al. 2002).

## Conclusions

The application of biomarker approach has proven to be a useful tool for assessing the environmental compatibility of chemical and physical treatments aimed at increasing the durability of wood.

The ecotoxicological investigations carried out using two model organisms (*D. polymorpha* and *T. fluviatilis*) to evaluate the toxicity of a Cu-based impregnating solution and a thermal modification applied in *Picea abies* wood panels exposed to freshwater, showed that:

- S-UC3 wood treatment, containing 0.25% Cu, resulted toxic for both species. The chemical treatment led to copper leaching, which caused 100% mortality of the nerite gastropods, more than 80% of the zebra mussels and resulted in physiological and biochemical stress signals in the survived organisms. Based on these results, modifications in the impregnation protocol of wood panels are therefore necessary before its application in natural freshwater environments.
- Thermally treated panels (TT) did not cause ecotoxicological effects in zebra mussels. In nerite gastropods, some oxidative and neurotoxic stress signals as well as an induction of metallothioneins were observed.
- NTC panels were not toxic to zebra mussels, whereas stress signals were revealed in nerite gastropods probably due to the leaching of natural toxic wood compound.
- The high mortality of *T. fluviatilis* in the control sample highlighted the low tolerance to aquarium conditions of this species; therefore, its use as a bioindicator organism in exposure experiments is not recommended.

## Data Availability

Data will be made available on reasonable request.
